# 17 β-Estradiol Oxidative Stress Attenuation and Autophagy-Induced Dopaminergic Neuroprotection

**DOI:** 10.22074/cellj.2019.5799

**Published:** 2018-11-18

**Authors:** Roya Varmazyar, Ali Noori-Zadeh, Farzad Rajaei, Shahram Darabi, Salar Bakhtiyari

**Affiliations:** 1 Student Research Committee, Qazvin University of Medical Sciences, Qazvin, Iran; 2 Department of Clinical Biochemistry, Faculty of Allied Medical Sciences, Ilam University of Medical Sciences, Ilam, Iran; 3 Cellular and Molecular Research Center, Qazvin University of Medical Sciences, Qazvin, Iran; 4 Department of Clinical Biochemistry, Faculty of Medicine, Ilam University of Medical Sciences, Ilam, Iran

**Keywords:** Autophagy, 17 β-estradiol, Parkinson’s Disease, *Ulk1*

## Abstract

**Objective:**

Degeneration of dopaminergic neurons in the substantia nigra of the brain stem is the main pathological aspect of Parkinson’s disease (PD). 17 β-estradiol (E2) has neuroprotective effects on substantia nigra, however, the underlined mechanism is not well-known. In this study, we evaluated the neuroprotective effects of E2 in the ovariectomized 6-hydroxydopamine- (6-OHDA) rat model of PD.

**Materials and Methods:**

In this experimental study, all animals were ovariectomized to avoid any further bias in E2 levels and then these ovariectomized rats were randomly assigned into three experimental groups (10 rats in each group): ovariectomized control group (OCG), ovariectomized degeneration group receiving 25 μg of 6-OHDA into the left corpus striatum (ODG), and ovariectomized E2 pretreatment group pretreated with 0.1 mgkg^-1^of 17 β-estradiol for three days prior to the destruction of corpus striatum with 6-OHDA (OE2PTG). The apomorphine behavioral test and Nissl staining were performed in all experimental groups. The expressions of Sequestosome-1 (*P62*), Unc- 51 like autophagy activating kinase (*Ulk1*), and microtubule-associated proteins 1A/1B light chain 3B (*Lc3*) genes were evaluated using reverse transcription- polymerase chain reaction (RT-PCR).

**Results:**

E2 administration reduced the damages to the dopaminergic neurons of the substantia nigra. The motor behavior, the number of rotations, and histological tests in the treatment group showed the cell survival improvement in comparison with the control groups indicating that E2 can inhibit the neurodegeneration. P62 and Lc3 were expressed in all experimental groups while *Ulk1* was not expressed in ODG group. Moreover, *Ulk1* was expressed after the treatment with E2 in OE2PTG group.

**Conclusion:**

E2 prevents neurodegeneration in dopaminergic neurons of the midbrain by over-expression of Ulk1 gene and augmenting the induction of autophagy.

## Introduction

Parkinson’s disease (PD) is a neurodegenerative motor disorder that affects 50% of elderly people over 85 years old (1). Although the etiology of PD is mainly unknown, some factors such as oxidative stress- induced mitochondrial damage, which in turn, increases the protein aggregations, is the molecular and cellular characterization of the disease. Moreover, several studies have indicated the relationship between autophagy deficiency and neurodegenerative diseases such as PD. In this regard, autophagy regulation has been considered a strategy for the treatment of neurodegenerative diseases.

Autophagy is the primary cellular catabolic program in response to cellular starvation and degradation of the damaged organelles. It is well accepted that 17 ß-estradiol (E2) has neuroprotective effects in many neurodegenerative diseases (2). E2 also plays a significant role in regulating the MAPK/ERK pathway (3). Epidemiological studies have demonstrated that men are more prone to PD by a ratio of 3:2 in comparison with women and estrogen affects the disease onset and the severity of the symptoms associated with the disease (4). In addition, it acts through the antioxidant system by increasing the brain blood flow (5). Some actions of estrogen such as the regulation of neurotransmitter function are mediated through genomic and non-genomic pathways (6).

In PD, the degeneration of dopaminergic neurons results from the accumulation of aggregated proteins caused by oxidative stress in the cell. In fact, autophagy mediated degradation of aggregated proteins and damaged organelles are disrupted, therefore, autophagy may be considered a therapeutic target. As the age increases, changes in the lysosomal activity can reduce the rate of autophagy in the neurodegenerative diseases (7, 8). However, the mechanism of its protective actions is still largely unknown, particularly in PD. In the present study, the mechanism of E2 in autophagymediated neuroprotection has been investigated in the rat model of PD. 

## Materials and Methods

### Animals 

In this experimental study, rats (female, Wistar) were maintained under a 12-12 hours light-dark condition at a controlled temperature of the animal laboratory. Water and food were available ad libitum for all of the animals. All ethical guidelines were followed in order to reduce the animal suffering. The study was conducted in accordance with the guidelines for working with experimental animals set by the Ethics Committee (Ethics code: IR.QUMS.REC.1395.67) of Qazvin University of Medical Sciences.

### Ovariectomy of animals 

In order to remove E2-producing gonads and hormonal cycle, the ovaries were both removed under sterile and aseptic conditions in all of the animals. After anesthetizing with a mixture of ketamine (100 mgkg^-1^, Sigma-Aldrich, Germany) and xylazine (5 mgkg^-1^, Sigma-Aldrich, Germany), the ovaries were removed after 1 cm cutting in the skin of the animal. Then, the skin of the ovariectomized rats was sutured.

### Development of Parkinson’s disease in ovariectomized rats 

For the development of Parkinson’s disease in the animal model, the ovariectomized rats were anesthetized by intraperitoneal injection of a mixture of Ketamine (100 mgkg^-1^) and Xylazine (5 mgkg^-1^). Their heads were then fixed in a stereotaxic device in accordance with the coordinates. The coordinates were set to 3 mm lateral to the left to cause a lesion, 4.5 mm abdominal from dura mater and +9.2 anterior- posterior to the interaural line. Incisor bar was also located 3.3 mm below the horizontal line. After fixing the animals’ head on the device, the skin can be exposed by removing hairs from the head using regular razors and scissors. After disinfecting the surgical site using Betadine, an incision was created parallel to the sagittal plane from a distance between the eyes to between the ears, and the scalp was sheared from the skull. After finding the coordinates, the bone for injection was drilled at low speed in order to protect the brain tissue from an injury. In the ovariectomized control group (OCG), stereotaxic surgery was performed on the rats and 5 µL of saline containing 0.2 % of ascorbate was injected into the left corpus striatum. In the ovariectomized degeneration group (ODG), 5 µL saline ascorbate 0.2% contained 25 µg of 6-OHDA was injected into the left corpus striatum of rats. The rats in ovariectomized E2 pretreatment group (OE2PTG) were pretreated with 0.1 mgkg^-1^ of 17 ß-estradiol (E8875, Sigma-Aldrich, Germany) for three days prior to the destruction of corpus striatum. After E2 pretreatment, the dura mater was exposed and 5 µL of saline ascorbate 0.2% contained 25 µg of 6-OHDA was injected into the left corpus striatum of rats using a 5-µl Hamilton syringe.

### Behavioral tests 

The behavioral test was performed on the rats in the three experimental groups before the surgery and four weeks afterward. Behavioral tests were carried out by intraperitoneal injection of apomorphine hydrochloride (Sigma-Aldrich, Germany) with a dose of 2.5 mgkg^-1^. Ten minutes before the surgery (baseline) rats were kept in a cylindrical transparent chamber made of glass with the diameter of 33 cm and the height of 35 cm. After injecting medication, the total 360-degree rotation was measured manually for 60 minutes at the intervals of 10 minutes. The number of contralateral (opposite the lesion site or to the right) and the number of ipsilateral rotations (toward the lesion site or to the left side) were considered the positive and negative numbers, respectively. The net number of the rotations was calculated after subtracting rotations from two directions.

### Nissl staining in experimental groups

By intraperitoneal injection of a mixture of ketamine (100 mgkg^-1^) and xylazine (5 mgkg^-1^), rats were anesthetized at the fourth week, i.e. after performing the behavioral tests. The rats were perfused using normal saline and formalin. After perfusion, the brain was removed from the skull. For neuronal counts, tissue blocks were provided from animals’ substantia nigra. Tissue sections with the diameter of 10 µm were made from the midbrain at intervals of 2.4 to 2.9 mm from the interaural point in accordance with the Paxinos atlas. The tissue sections were Nissl-stained with Cresyl violet solution (0.1%). The neurons in the dense part of substantia nigra were counted in sections aligned with 4 levels of Paxinos atlas (i.e., 2.96, 3.2, 3.8, and 4.2) as compared to the center of interaural line with the magnification of (×200, ×100). At each level, at least two sections were counted and the neurons with the cytoplasmic domain were also counted.

### Gene expression analysis

The total RNA was isolated from the striatum of each animal using Ambion kit (Invitrogen, USA) following the manufacturer’s instructions. Each sample of the isolated RNA was further treated with DNase I enzyme (Invitrogen, USA). The yield and quality of the total RNA were assessed using absorbance ratio at (260 nm/280 nm) using spectrophotometry and denaturing agarose gel electrophoresis. The reverse transcription-polymerase chain reaction (RT-PCR) was performed using the RevertAid first strand cDNA synthesis kit (Fermentas, Lithuania) according to the manufacturer’s instructions. Meanwhile, glyceraldehyde-3-phosphate dehydrogenase (*Gapdh*) was used as an internal control gene. The primers have been shown in Table 1. 

**Table 1:** The sequence of the primer pairs and corresponding amplicon sizes that have been used in this study


Gene	Primer (5′-3′)	Amplicon size (bp)

*Ulk1*	F: AAGGATTGGAAGGGTGGAGG	195
	R: ATGGGAAGGATGGTGGCTG	
*Lc3*	F: TGTTAGGCTTGCTCTTTTGG	219
	R: GCAGAGGAAATGACCACAGAT	
*Gapdh*	F: ATCTGACATGCCGCCTGGAG	154
	R: AAGGTGGAAGAATGGGAGTTGC	
*P62*	F: TCCTACAGACCAAGAATTATGAC	232
	R: TTCTCATGCACTTTCCTACTG	


### Statistical analysis

All data were expressed as mean ± SEM (any exception is mentioned). Moreover, one-way ANOVA was used for the results obtained from investigating apomorphineinduced rotational behavior in two periods (i.e., before and 4 weeks after surgery). One-way ANOVA was used to evaluate the mean neurons in the dense part of substantia nigra and multiple post-hoc comparisons were performed by Tukey’s test between the groups. In addition, Microsoft Excel (2017) was used in order to draw the diagrams. P<0.05 was considered as a significant statistical difference. 

## Results

### Apomorphine-induced rotational behavior test

**Fig.1:**
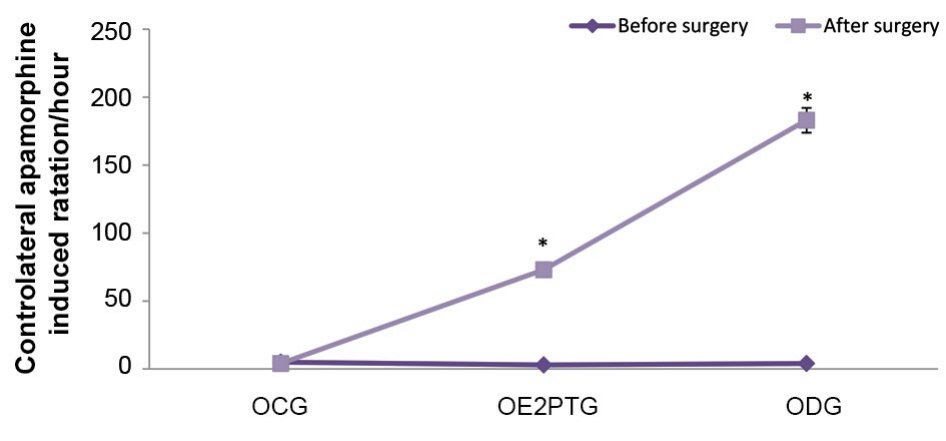
Before the surgery, there was no significant difference among the OCG, ODG, and OE2PTG groups in the rotations (P<0.05). OCG; Ovariectomized control group, ODG; Ovariectomized degeneration group, OE2PTG; Ovariectomized E2 pretreatment group, and *; Indicates a significant difference between each experimental group with the OCG group.

The behavioral test was performed at the 1^st^ and 4^th^ weeks of the surgery. The results indicated that the rotations before the surgery were 5 ± 0.36, 3 ± 0.39, and 4 ± 0.42 (mean ± SEM) meanwhile at 4 weeks post-surgery the rotations were 4 ± 0.44, 73.53 ± 1, and 183 ± 4.78 for the OCG, OE2PTG and, ODG groups, respectively. The rotation results in the ODG group suggested the verification of substantia nigra degradation in the animal model. Moreover, E2 reduced the damage to the dopaminergic neurons of substantia nigra which was characterized by improving the motor behavior and reducing rotations in the OE2PTG group in comparison with ODG group. There was a significant difference (P<0.05) of rotations between the OCG and ODG groups (Fig .1). Before the surgery, there was no significant difference among the OCG, ODG, and OE2PTG groups in the rotations (Fig .1). 

### Nissl staining of substantia nigra

**Fig.2:**
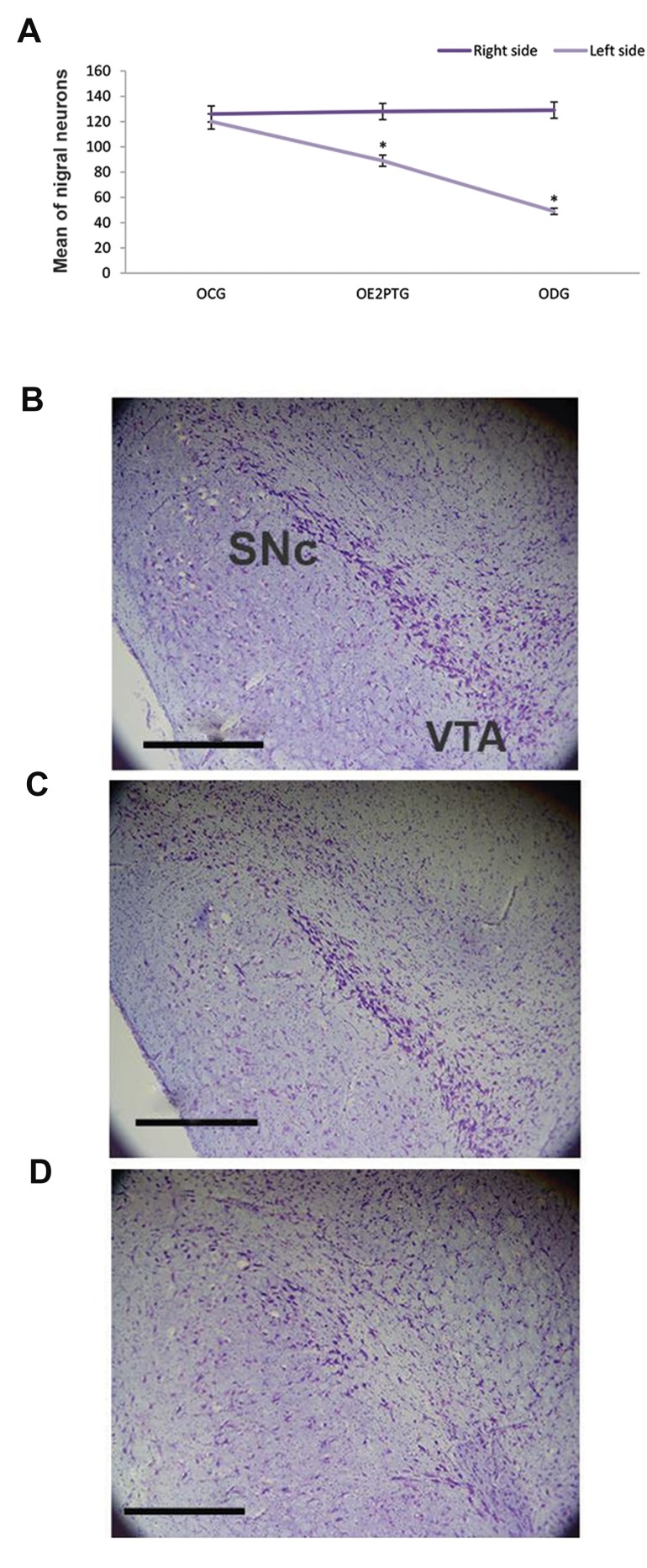
Neuronal counts in the substantia nigra. A. The means of nigral neurons in the left and right sides of the three experimental groups have been shown. On the right side, there were no significant differences among the groups. However, for the left side, a significant difference was observed for all groups (P<0.05). Neurons in substantia nigra in the left side of the experimental groups with Nissl staining for B. OCG group, C. OE2PTG group, and D. ODG group. Abundant neurons existed in the substantia nigra and ventral tegmental area of OCG and OE2PTG groups. In contrast, the number of neurons was progressively decreased in substantia nigra ipsilateral to 6-OHDA injection in ODG group. OCG, ODG, ovariectomized E2 pretreatment group (OE2PTG), substantia nigra pars compacta (SNc), ventral tegmentum area (VTA) (scale bars: 200 µm). OCG; Ovariectomized control group, ODG; Ovariectomized degeneration group, and *; Shows the statistically significant difference in OCG group (P<0.05).

The midbrain was separated and after preparing the tissue block, neuronal counts were done using Nissl staining. The results indicated that the means ± SEM for the neurons in the right (normal area) substantia nigra for the OCG, OE2PTG, and ODG groups were 126 ± 3.18, 128 ± 2.73, and 129 ± 2.64, respectively; suggesting that there were no significant differences among the groups. Moreover, the means ± SEM for neurons in the left (degenerated area) substantia nigra for the OCG, OE2PTG, and ODG groups were 120 ± 2.19, 89 ± 1.68, and 49 ± 1.67, respectively suggesting a significant (P<0.05) reduction of neurons in the groups as compared to control group (Fig .2). Progressive degeneration of the nigral dopaminergic neurons after 6-OHDA administration was observed in ODG group (Fig .2A). In ODG group, the number of neurons was statistically less than OCG group suggesting the degeneration of neurons in substantia nigra by 6-OHDA (Fig .2). In OE2PTG group (Fig .2B), 17 ß-estradiol prevented the neuronal degeneration of substantia nigra in OE2PTG group and fewer neurons degenerated in comparison with the OCG group (Fig .2C). 

### Gene expression analysis 

**Fig.3:**
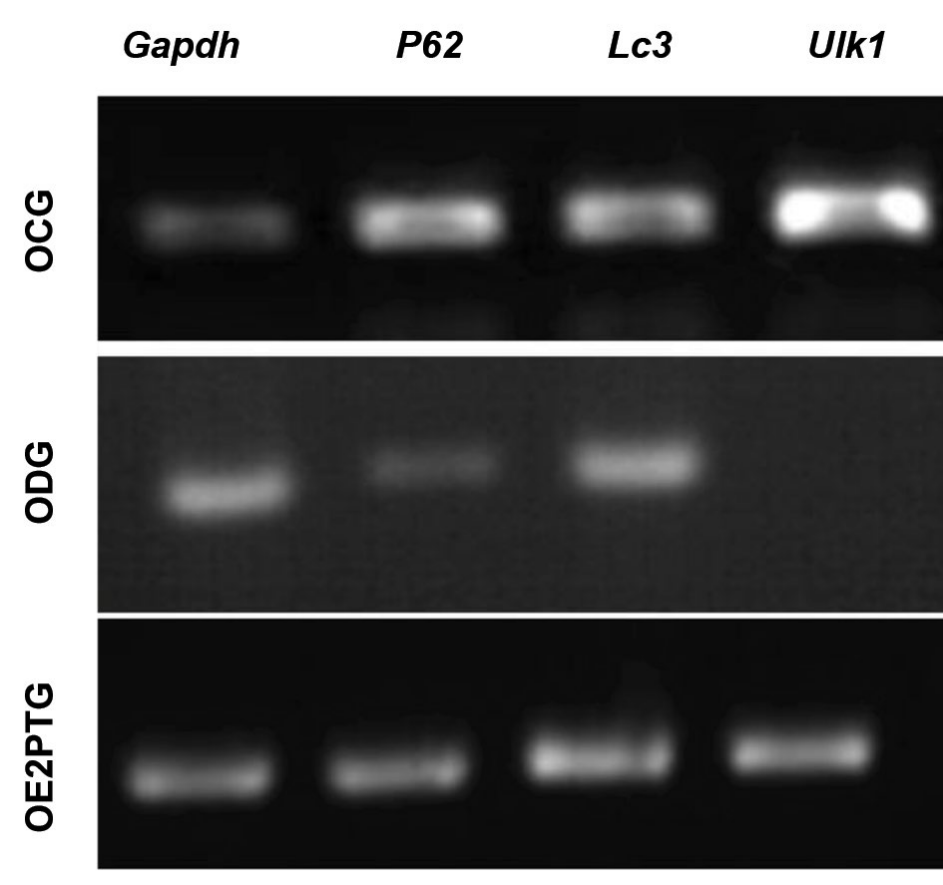
Gene expression results. The P62 and Lc3 expressed in all groups, while *Ulk1* was expressed only in ovariectomized degeneration (ODG) group. In ovariectomized rats pretreated with 17 ß-estradiol before 6-hydroxydopamine injection (OE2PTG), *Ulk1* was overexpressed. *Gapdh* was used as an internal control which was expressed in all groups. OCG; Ovariectomized control group.

The results of *P62, Ulk1*, and *Lc3* gene expression analyses in the three experimental groups indicated that *P62* and *Lc3* genes expressed in all groups while Ulk1 was only expressed in ODG group. In OE2PTG group after receiving E2, *Ulk1* was overexpressed (Fig .3). *Gapdh* was used as an internal control expressed in all groups.

## Discussion

In the present experimental study, 17 ß-estradiol i. Improved the motor behavior and reduced apomorphineinduced rotational behavior, ii. Reduced the degeneration of substantia nigra neurons which was induced by the neurotoxic effects of 6-OHDA, and iii. Overexpression of *ULK1* inhibited by 6-OHDA. In this study, 6-OHDA injections caused behavioral and tissue changes in accordance with PD model development. This model for PD is the most common pre-clinical model that has been well known due to its effects on the nigrostriatal dopaminergic system. 6-OHDA model caused molecular changes in the substantia nigra, which is most similar to PD characteristics in humans. The biological functions of estrogen are mediated by binding to the estrogen receptor -a and estrogen receptor-ß; by which estrogen has a slow genomic mechanism that protects the cells against apoptosis and inflammatory reactions and regulates the growth factors and neurotrophins and contributes to the formation of synapses. 

Studies have also suggested that ovarian removal can cause significant behavioral changes in apomorphineinduced in animals (9). Such changes can be due to the reduced number of dopaminergic neurons in substantia nigra (10). In addition, these neurotransmitter changes following the removal of the gonads can justify the nervous system disorder in women after the menopause. Another study in ovariectomized rats indicated the ability of estrogen to increase the dopamine absorption in the nigrostriatal dopaminergic system (11). In a study conducted in monkeys, it was observed that more than 30% of dopaminergic neurons in substantia nigra were disappeared 30 days after ovariectomy and estrogen prevented the degeneration of neurons within 10 days (12), however, they did not explore the underlying mechanism. In a study conducted *in vitro* model of PD, it was observed that estrogen is able to prevent the cell apoptosis against 6-OHDA toxicity by activating anti-apoptotic proteins and inhibiting pro-apoptotic proteins (13). Yet, they did not investigate the other estrogen pleiotropic effects. Studies have shown that 17ß-estradiol mediates its effect through the dopamine receptors (14). For the treatment of neurological diseases, cell and gene therapy along with various methods for the differentiation of mesenchymal stem cell and their differentiation into the neurons have been widely used (15-18). Moreover, epigenetic alteration and sex hormone therapy may be the other available treatment options as well. Indeed, studies have also indicated that the sex hormones are effective in the treatment of other neurodegenerative diseases (19) as we showed earlier. Consistent with our study, it has been recently shown that 17 ß-estradiol can regulate autophagy (20).

Macroautophagy is a conserved protein degradation mechanism in which the cargo is surrounded by autophagosome and then fused with the lysosome. In the initiation phase of autophagy, the first step is the formation of autophagosome. ULK1 as an upstream protein starts the process of autophagy and is regulated by signals such as mTOR, AMP-activated protein kinase (AMPK), and glycogen synthase kinase 3 (GSK3) (21). Under the normal conditions, mTOR is phosphorylated and negatively regulates the complexes such as ULK1, ULK2, ATG101, ATG13, and FIP200. As mTOR is inhibited, ULK1 activation results in activation of ATG13 and FIP200 upon the initiation of autophagy. The deficiency in autophagy can cause neurodegenerative diseases such as Alzheimer’s disease, Parkinson’s disease, and amyotrophic lateral sclerosis (22, 23). In PD, phosphorylated a-synuclein is fibrillated and accumulated known as Lewy bodies (24). The ULK1 has been observed in Lewy bodies. Evidence suggests that the downstream protein, LC3, contributes to Lewy body formation. Phosphatidyl ethanolamineconjugated form of LC3 (LC3II) is bound to the internal surface of autophagosome and acts as a clasp for the cargo receptors such as P62 (25). These results indicate that autophagy-lysosome system plays a significant role in the pathogenesis of PD and Lewy body formation. In the present study, 17 ß-estradiol increased the expression of ULK1 in animals with PD. In another study, 17 ß -estradiol prevented osteoblast cell death by activating autophagy and ER-ERK-mTOR and expressing ULK1 and Beclin-1 (20). ULK1 plays a significant role in the bingeing of the autophagy process. The deficiency in autophagy can also cause the abnormal protein accumulations and damage to the organelles in neurodegeneration. Since some PD models can impair mitochondrial functions, deficiency in controlling the mitochondrial quality plays a crucial role in the pathogenesis of PD. The studies have shown that selective degradation of damaged mitochondria is a part of an important homeostasis pathway for controlling the organelles quality and mitophagy (mitochondrial autophagy) playing a vital role in mitochondrial decomposition and maintaining dopaminergic neurons. 

On the other hand, protein accumulation as a cellular pathology has been observed in many neurodegenerative diseases including PD. In this context, autophagy is considered one of the major proteolytic systems which can maintain the homeostasis of the cellular proteins. ULK1 is required to form autophagosomes in mammalian cells. It has been proven that ULK1 and 2 are necessary for autophagy. *LC3* is one of the autophagic genes that its product accumulates in the autophagosome membrane and is considered an autophagy marker (25). ATG101 is a binding protein for ATG13 which is a part of ATG1/ULK1 serine-threonine kinase and is required for autophagy induction. The ULK1 complex contains ATG13 and FIP200 which are required for autophagy initiation. The interaction between ATG101 and ATG13 is important for the stability and phosphorylation of ATG13 and ULK1. Therefore, the lack of *ULK1* expression leads to the disturbance in the initiation of autophagy. 

## Conclusion

In this study, the administration of 17 ß-estradiol led to *Ulk1* overexpression and regulating autophagy accompanied by the improvement in behavioral and tissue of animal model of PD. 
